# Circulating Innate Lymphoid Cells Exhibit Distinctive Distribution During Normal Pregnancy

**DOI:** 10.1007/s43032-021-00834-6

**Published:** 2022-01-05

**Authors:** Yiran Zhao, Yajie Zhu, Xi Chen, Hui Lin, Ningxin Qin, Zhiyang Zhou, Han Liu, Yanhui Hao, Chengliang Zhou, Xinmei Liu, Li Jin, Jianzhong Sheng, Hefeng Huang

**Affiliations:** 1 The International Peace Maternity & Child Health Hospital, School of Medicine, Shanghai Jiao Tong University, Shanghai, 200030 China; 2 Shanghai Key Laboratory of Embryo Original Diseases, Shanghai, 200030 China; 3 Hospital of Obstetrics and Gynecology, Fudan University, Shanghai, 200010 China; 4 Department of Pathology and Pathophysiology, School of Medicine, Zhejiang University, Zhejiang, 310058 China

**Keywords:** Innate lymphoid cells, Innate immunity, Pregnancy, Sex hormones, Estradiol, Progesterone

## Abstract

Over the past decades, the investigation of innate lymphoid cells (ILCs) has revealed their significance in successful pregnancy. Sex hormones, such as estradiol and progesterone, show specific changes during pregnancy and modulate both adaptive and innate immune systems. ILC subset distribution in peripheral blood of pregnant women and its potential association with sex hormone levels have not been well revealed. Peripheral blood was obtained from healthy non-pregnant, early-pregnant, and late-pregnant women. Radioimmunoassay was performed to measure plasma estradiol and progesterone levels. The levels of type 1 ILCs (ILC1s), type 2 ILCs (ILC2s), type 3 ILCs (ILC3s), and total ILCs as well as estrogen and progesterone receptors of ILC2s in peripheral blood were analyzed using flow cytometry. The proportion of total ILCs and distribution of ILC subsets in peripheral blood changed dynamically during pregnancy. Compared to non-pregnant women, late-pregnant women displayed significantly higher proportion of circulating ILCs, among which ILC2s accounted for the majority in late-pregnant women while a smaller part in others, and ILC3s displayed the opposite. Plasma estradiol and progesterone levels elevated while pregnancy proceeded and the expression of their receptors in ILC2s increased consisted with the proportion of circulating ILC2s. Our work first observed the existence of progesterone receptors in human circulating ILC2s and revealed the distribution pattern of circulating ILC subsets and their interrelation with plasma sex hormone levels during pregnancy. Our results suggested that the estradiol and progesterone levels might partly influence the distribution of circulating ILC subsets and implied the interplay between circulating ILCs and pregnancy.

**Supplementary Information:**

The online version contains supplementary material available at 10.1007/s43032-021-00834-6.

## Introduction

Successful pregnancy relies on the sophisticated balance of the immune system which maintains tolerance to the semiallogeneic fetus, while sustains innate and adaptability to meet challenges [1–3]. Recent studies have shown that T cells and their subgroups play a key role in preserving fetal development [4–7]. While the adaptive immune system plays an important role, the innate immune system also matters [1, 8–12]. Innate lymphoid cells (ILCs) are a developing family of innate immune cells lacking lineage markers for T cells, B cells, myeloid and dendritic cells, monocytes and macrophages, mast cells, and stem cells and mirror the functions of T cells. Natural killer (NK) cells were the only known ILCs for much of past years; however, several new ILC populations have been identified recently. Identical to lymphocytes, ILCs develop from the common lymphoid progenitor, but distinct transcription factors suppress the lymphocyte fates and lead to the generation of the different subsets of ILCs. According to the functions parallel to those of T cells, ILCs can be generally classified into two types: helper type ILCs and cytotoxic ILCs. Helper type ILCs include type 1 ILCs (ILC1s), type 2 ILCs (ILC2s), and type 3 ILCs (ILC3s), which are respectively the innate counterparts of CD4^+^ T helper (Th) 1, Th2, and Th17 cells. As a conventional group of innate cells, NK cells are termed as cytotoxic ILCs recently, mirror the functions of CD8^+^ cytotoxic T cells [13, 14]. Over the last decade, ILCs have been identified not only in human mucosal tissues, but also non-mucosal tissues, such as peripheral blood [15].

More recently, the significance of ILCs in successful pregnancy has been revealed [12, 16–20]. ILCs have been identified in maternal-fetal compartments in early weeks of pregnancy in human as well as mice [8], among which, NK cells are the most conspicuous during the first trimester of pregnancy in human decidua [21], while ILC2s were just a small proportion [12]. In the process of pregnancy, NK cells start to decline and ILC2s gradually become the most abundant subsets in human decidua of late pregnancy [19]. Moreover, ILC2 is the most abundant ILC subset in murine uterus [22], indicating their underline effect in maintaining normal pregnancy. The dynamic changes of ILC subsets in human decidua and murine uterus suggest an important role of ILCs in maintaining healthy pregnancy, and an abnormal increasing of ILC subsets may lead to a pathologic pregnancy, such as preterm labor [19, 23].

Hormones are also necessary for maintaining the adequate environment for successful pregnancy. Particularly, sex hormones are of great importance, the most studied are progesterone and estrogens [24–29]. Estradiol (E2), the predominant and most biologically active form, belongs together with estrone (E1) and estriol (E3) to estrogens [30]. During three trimesters of pregnancy, progesterone and estradiol levels elevate as pregnancy progresses, which adapt to the physiological changes [24, 25]. Progesterone and estradiol have been reported to modulate both adaptive and innate immune cells in human and mice, such as T cells, B cells, macrophages, dendritic cells, and NK cells [31–41]. As previously described, estradiol has an estrogen receptor α (ERα)-selective agonist potency [42], which mainly exists in the nucleus, and can regulate ILC2 accumulation in murine uterus as well as modulate expression of a series of genes through highly expressed ERα [43].

Both helper type ILCs and NK cells as well as sex hormones, as mentioned above, are of great importance for healthy pregnancy. Compared to NK cells, the variation of circulating helper type ILCs during normal pregnancy remains unrevealed. Our study aimed to explore the dynamic changes of helper type ILC subsets before and during normal pregnancy, and whether plasma sex hormone levels correlate with ILC distribution in human peripheral blood. Using flow cytometry, we investigated the proportion and absolute counts of circulating helper type ILC subsets in non-pregnant and pregnant women as well as their correlation with increasing plasma progesterone and estradiol levels. Generally, our study is the first to discover the variation of circulating helper type ILC distribution in different gestation and observed the expression of progesterone receptor in human circulating ILC2s. Our work provides new insights into the potential mechanisms of sex hormones influencing ILCs in peripheral blood and indicates the physiological functions of circulating ILCs during pregnancy.

## Methods and Materials

### Study Population and Sampling

All subjects were Chinese pregnant women from The International Peace Maternity and Child Health Hospital (Shanghai, China) between April 2020 and January 2021. Twenty-five non-pregnant women, 25 early-pregnant (6–12 weeks) women, and 25 late-pregnant (29–42 weeks) women were all from outpatient department. Ethical approval for the study was obtained from the Ethics Review Committee/Institutional Review Board (GKLW 2017–01). Written informed consent was obtained from each participant. Only healthy women who were 25–35 years old were included, and pregnant women were all with a normal pregnancy. Previous pregnancies and deliveries must have been uncomplicated. Exclusion criteria were women who had a history of preeclampsia or gestational hypertension, diabetes, thyroid disease, anemia, intrahepatic cholestasis of pregnancy, spontaneous abortion, or preterm delivery. Gestational age was estimated by the crown-rump length, which measured by an ultrasound scan. Samples of peripheral blood from each group were included in the study, and there were no overlaps among three groups.

### Isolation of Plasma and Peripheral Blood Mononuclear Cells (PBMCs)

Venous blood samples of non-pregnant and pregnant women were collected using routine tubes, respectively. After centrifuged at 4 °C for 15 min at 2000 *g*, plasma was obtained and stored at −80 °C for the measurement of estradiol and progesterone. Using Ficoll-Hypaque density gradient centrifugation at 20 °C for 30 min at 900 *g*, peripheral blood mononuclear cells (PBMCs) were obtained from 1 ml peripheral blood and counted by Cellometer Auto 1000 (Nexcelom Bioscience, Lawrence, USA), then followed by analyses of ILC subsets using flow cytometry. Analyses were performed on fresh samples.

### Flow Cytometry

To identify ILC subsets, the following antibodies to human proteins were stained with PBMCs: FITC-conjugated anti-CD5 (UCTH2), anti-CD16 (3G8), anti-CD56 (NCAM16.2), anti-CD11c (B-ly6), anti-CD34 (581), anti-TCRɑβ (T10B9.1A-31), APC-Cy7-conjugated anti-CD45 (2D1), BV421-conjugated anti-CRTH2 CD294 (CRTH2; BM16), APC- or APC-R700-conjugated anti-CD117 (C-Kit; YB5.B8), and R Phycoerythrin-Cyanine 7 (PE-Cy7)-conjugated anti-CD127 (HIL-7R-M21; all from BD Biosciences, Mountain View, USA); FITC-conjugated anti-CD3ε (HIT3a), anti- CD4 (RPA-T4), and anti-CD11b (M1/70; all from BioLegend, San Diego, USA); FITC-conjugated anti-CD14 (61D3), anti-CD19 (HIB19), anti-CD123 (6H6), anti-TCRγδ (B1.1), and anti-FcεRIɑ (AER-37 (CRA1); all from eBioscience, San Diego, USA).

For measurement of intranuclear estrogen and progesterone receptors, PBMCs were isolated directly ex vivo, firstly stained with antibodies to surface antigens, fixed and permeabilized using Foxp3/Transcription Factor Staining Buffer Set (eBioscience, San Diego, USA) according to the manufacturer’s instructions, and secondly stained with phycoerythrin (PE)-conjugated anti-ERα (ab209288, Abcam, Cambridge, UK) and Alexa Fluor 647-conjugated anti-progesterone receptor (D8Q2J, Cell Signaling Technology, Danvers, USA).

Samples were acquired on BD FACSCelesta flow cytometer instrument (BD, Franklin Lakes, USA) and analyzed using FlowJo software, version 10.7.0 (BD, Franklin Lakes, USA).

### Radioimmunoassay (RIA)

The plasma concentrations of estradiol and progesterone in non-pregnant, early-pregnant, and late-pregnant women were measured by radioimmunoassay (RIA) using XH6080 radio-immune analyzer (Xi’an Nuclear Instrument Factory, Xi’an, China) and following the manufacturer’s instructions (Beijing North Institute of Biotechnology Co., Ltd).

### Statistical Analysis

Statistical analyses were performed with GraphPad Prism software, version 8.4.3 (GraphPad Software Inc., CA, USA). The Kruskal-Wallis tests, one-way ANOVA, and chi-square tests were employed for comparisons among three study groups, and Mann-Whitney test was used for comparisons between early-pregnant and late-pregnant groups. To analyze the correlations between estradiol as well as progesterone levels and ILC2 proportion, Spearman’s correlation analysis was performed. *P*-values less than 0.05 (*P* < 0.05) were considered significant. Graphs were plotted using GraphPad Prism software, version 8.4.3 (GraphPad Software Inc., CA, USA).

## Results

### Patient Characteristics

Seventy-five healthy women who were 25–35 years old were included, of which 25 were non-pregnant, 25 were early-pregnant, and 25 were late-pregnant (Table 1). No significant difference was noted in age, pre-pregnancy body mass index (BMI), and times of previous induced abortions among three study groups. Since pregnant women employed periodical inspection where blood sample were collected, the gestational age of early-pregnant and late-pregnant women was relatively concentrated in a certain interval.

**Table 1:** Participant characteristics

Characteristic	Non-pregnant	Early-pregnant	Late-pregnant	*P* value
*N*	25	25	25	
Age, mean (*SD*), years	30 (3)	29.72 (2.31)	30.36 (2.66)	0.6990
Pre-pregnancy BMI, mean (*SD*), kg/m^2^	20.12 (1.32)	20.21 (1.48)	20.39 (2.20)	0.8511
Gravidity, *n* (%)				< 0.001***
0	7 (28)	0 (0)	0 (0)	
1	8 (32)	4 (16)	15 (60)	
≥2	10 (40)	21 (84)	10 (40)	
Parity, *n* (%)				0.0176*
0	15 (60)	8 (32)	19 (76)	
1	4 (16)	9 (36)	5 (20)	
≥2	6 (24)	8 (32)	1 (4)	
Previous induced abortions, *n* (%)				0.2607
0	10 (40)	11 (44)	16 (64)	
1	11 (44)	10 (40)	4 (16)	
≥2	4 (16)	4 (16)	5 (20)	
Gestational age, median (Q1, Q3), weeks		6 (6, 6)	39.14 (38.79, 39.79)	< 0.001***

Comparisons of characteristics among non-pregnant, early-pregnant, and late-pregnant women were assessed using Kruskal-Wallis tests or one-way ANOVA or Mann-Whitney test for continuous variables and chi-squared tests were used for categorical variables. *P*-values less than 0.05 (*P* < 0.05) were considered significant. *P < 0.05 and ***P < 0.001. *BMI* body mass index, *SD* standard deviation, *Q1* the first quartile, *Q3* the third quartile

### Late Pregnancy Displays the Highest-Circulating ILC Proportion in Human Peripheral Blood

**Fig. 1:**
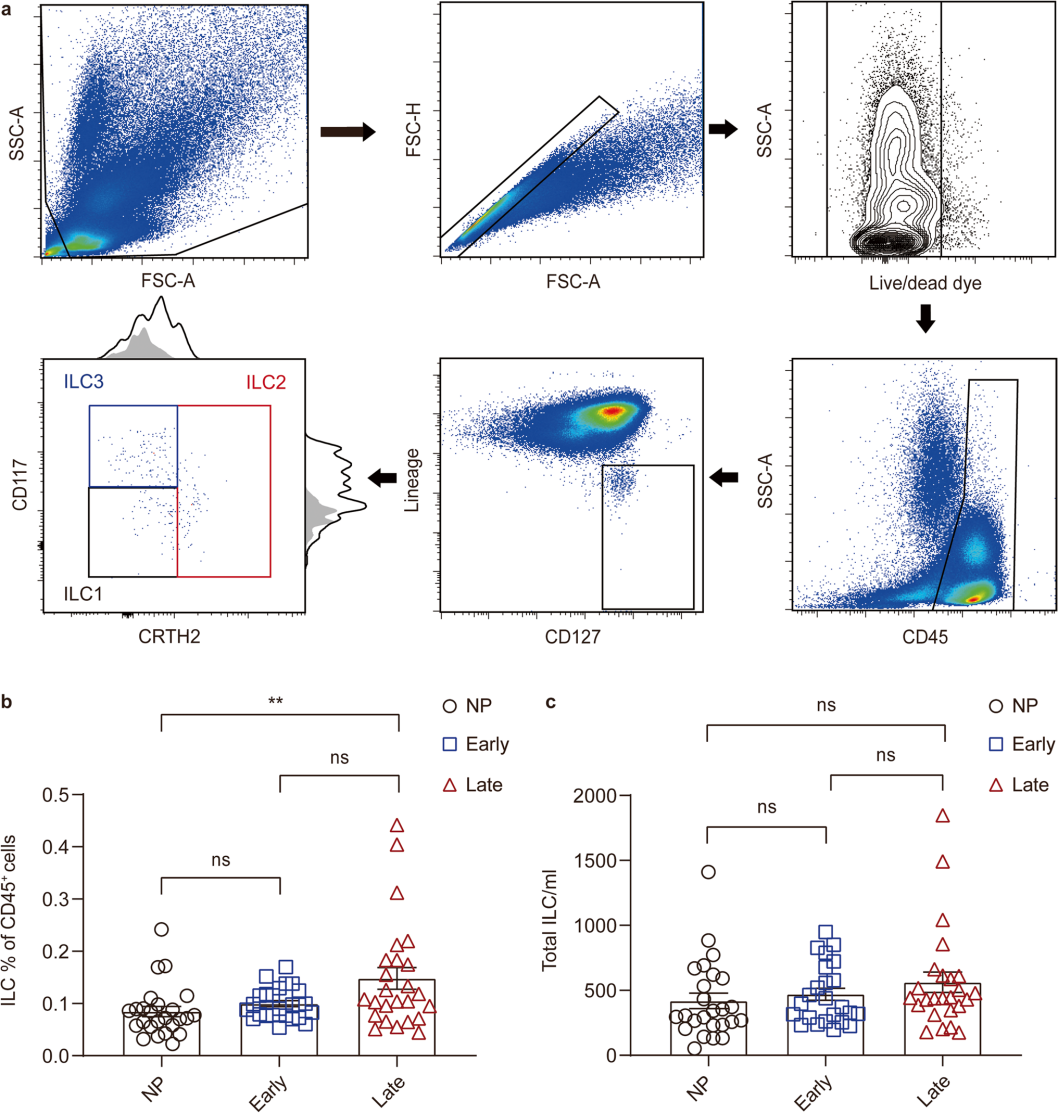
Late pregnancy displays the highest circulating ILC proportion in human peripheral blood. **a** Flow cytometry gating strategies for the identification of peripheral blood ILC subsets. ILCs are defined as CD45^+^Lin^−^ (including CD3ε, CD4, CD5, CD14, CD19, CD56, CD11c, CD11b, CD34, CD123, TCRɑβ, TCRγδ, and FcεRIɑ) CD127^+^ cells. The distribution of ILC1 (CD117^−^CRTH2^−^; black), ILC2 (CRTH2^+^; red) and ILC3s (CD117^+^CRTH2^−^; blue) were determined. Histograms represent expression analysis with the FMO as grey area and the stained sample as line. Comparison of circulating ILC percentage in CD45^+^ cells (**b**) and absolute counts (**c**) in non-pregnant, early-pregnant, and late-pregnant women. *NP*, non-pregnant women. Data are shown as means ± SEMs and were analyzed by Kruskal-Wallis tests. Each point indicates an individual. **P < 0.01. *ns*, not significant

ILCs are a discrete population of CD45^+^ cells defined as lineage-negative (Lin^−^) cells with the expression of the ɑ-chain of the IL-7 receptor (CD127) [19, 44]. As previously described, a combination of Lin markers, comprised of CD3ε, CD4, CD5, CD14, CD16, CD19, CD56, CD11c, CD11b, CD34, CD123, TCRɑβ, TCRγδ, and FcεRIɑ, was used in this study [44–46]. The gating strategy used to identify circulating ILC subsets is shown in Fig. 1a.

To explore the physiological changes of circulating ILCs in non-pregnant and pregnant women of different trimesters, we first detected the percentages of total ILCs within CD45^+^ cells in peripheral blood. We found that the level of total ILCs was significantly higher in late-pregnant women than the non-pregnant group (*P* = 0.0053), while it was comparable to early-pregnant women (*P* = 0.6975) (Fig. 1b). Then we analyzed the absolute counts of ILCs per milliliter of blood in three study groups. No statistically significant difference in ILC absolute counts was found among three study groups (*P* > 0.05) (Fig. 1c).

### ILC Subsets Show Distinctive Distribution in Peripheral Blood Before and During Pregnancy

To further determine which group of circulating ILCs has changed during pregnancy, we conducted an analysis of the proportion and phenotypes of ILC subsets. ILC1s and ILC3s were respectively defined as CD117^−^CRTH2^−^ and CD117^+^CRTH2^−^ cells, and CRTH2^+^ cells were considered as ILC2s [47] (Fig. 1a).

**Fig. 2:**
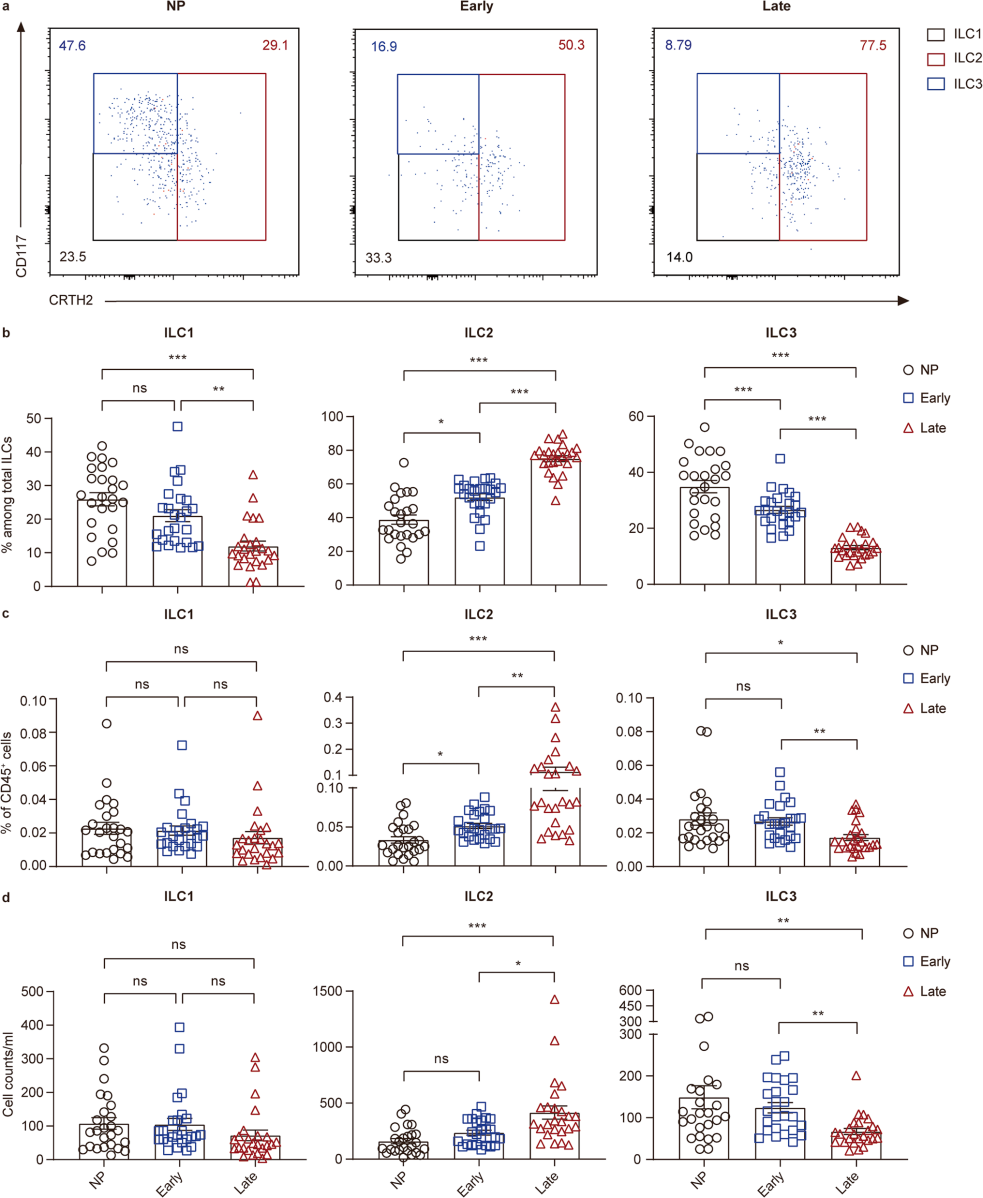
ILC subsets distribute dynamically in peripheral blood of non-pregnant women and pregnant women of different trimesters. **a–d** Analysis results for ILC subsets in peripheral blood samples of non-pregnant, early-pregnant, and late-pregnant women. **a** Representative flow cytometry plots are shown in which numbers indicate the frequency of flow cytometric events. Comparison of proportion of ILC subsets within total ILCs (**b**), ILC subset percentage in CD45^+^ cells (**c**) and absolute counts per milliliter of blood (**d**) in peripheral blood of non-pregnant women (black circles), early-pregnant women (blue squares), and late-pregnant women (red triangles). Data are shown as means ± SEMs and were analyzed by Kruskal-Wallis tests. Each point indicates an individual. *P < 0.05, **P < 0.01, and ***P < 0.001. *ns*, not significant

The analysis of ILC subsets using flow cytometry demonstrated that ILC2s accounted for the majority of total circulating ILCs (CD45^+^Lin^−^CD127^+^) in late-pregnant women while occupied smaller parts in early-pregnant (*P* < 0.001) and non-pregnant women (*P* < 0.001) (Fig. 2a). Furthermore, the percentage of ILC2 in total ILCs in early-pregnant women was statistically higher than non-pregnant women (*P* = 0.0364) (Fig. 2a and b). ILC2 proportion in circulating CD45^+^ cells shared the same variation trend as that in total ILCs that the ILC2 percentage was much higher in late-pregnant women than that in early-pregnant (*P* = 0.0087) and non-pregnant women (*P* < 0.001), while the subtle difference was found between early-pregnant and non-pregnant women (*P* = 0.0305) (Fig. 2c). We further evaluated the absolute counts of ILC2s per milliliter of blood in three study groups. It turned out that the count of ILC2 per milliliter of blood in late-pregnant women was statistically higher than that in early-pregnant (*P* = 0.0403) and non-pregnant women (*P* < 0.001) (Fig. 2d). Although there was no significant difference in circulating ILC2 counts between early-pregnant and non-pregnant women (*P* > 0.05), the increasing trend still existed.

Circulating ILC3s in late-pregnant women accounted for a much smaller part of total ILCs than early-pregnant (*P* < 0.001) and non-pregnant women (*P* < 0.001), statistical difference was also observed between early-pregnant and non-pregnant women (*P* < 0.001) (Fig. 2a and b). The absolute count of ILC3s per milliliter of blood and the percentage in CD45^+^ cells were much lower in late-pregnant women than those in early-pregnant (*P* = 0.0016; *P* = 0.0021) and non-pregnant women (*P* = 0.0026; *P* = 0.0105), whereas there was no difference between non-pregnant and early-pregnant women (both *P* > 0.05) (Fig. 2c and d). Although, same as ILC3s, circulating ILC1s in late-pregnant women accounted for a smaller proportion of total ILCs than early-pregnant (*P* < 0.001) as well as non-pregnant women (*P* < 0.001) (Fig. 2a and b). There was no difference found in neither ILC1 percentages in CD45^+^ cells nor absolute cell counts among the three study groups (Fig. 2c and d).

Taken together, these results reveal the dynamic changes of ILC subset distribution during pregnancy and suggest that these changes are associated with different trimesters of pregnancy.

### Elevating Estradiol and Progesterone Levels During Pregnancy Are Correlated with the Increasing Level of Circulating ILC2s

**Fig. 3:**
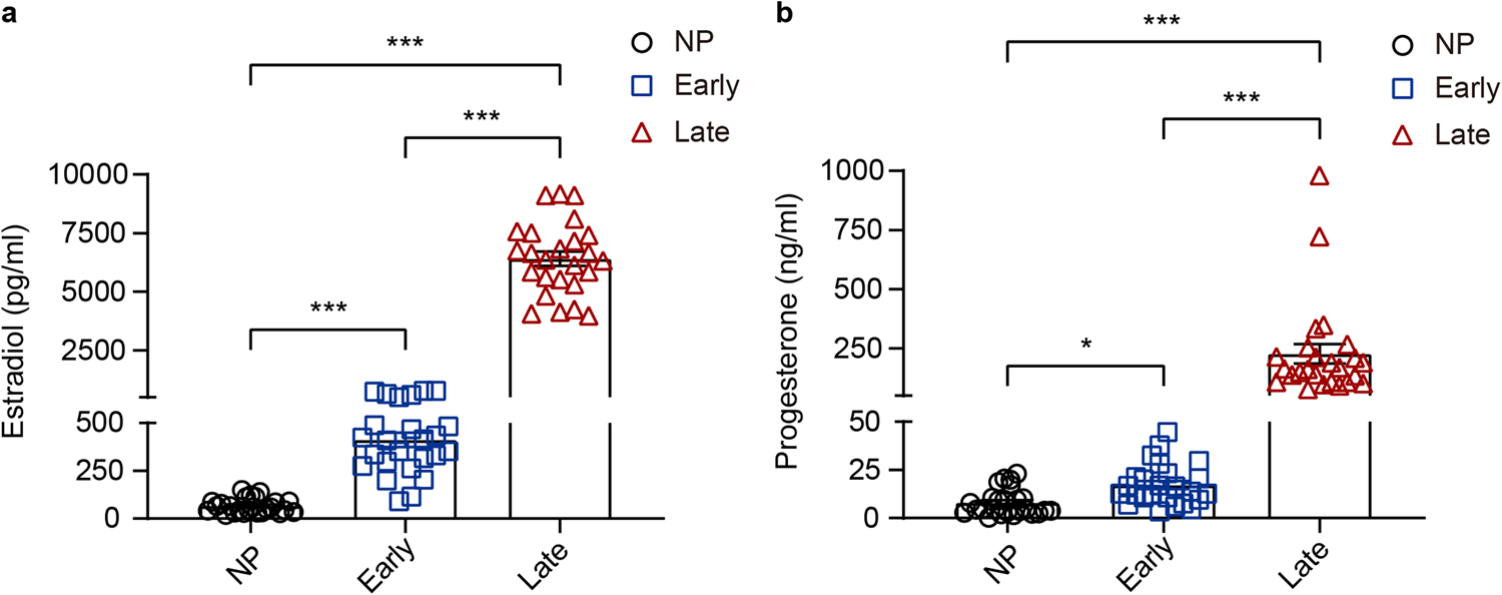
Plasma estradiol and progesterone levels increase as pregnancy proceeds. Comparison of concentrations of estradiol (**a**) and progesterone (**b**) in plasma of non-pregnant women (black circles), early-pregnant women (blue squares), and late-pregnant women (red triangles). Data are shown as means ± SEMs and were analyzed by Kruskal-Wallis tests. *P < 0.05 and ***P < 0.001

Next, we evaluated plasma estradiol and progesterone levels of non-pregnant and pregnant women of different trimesters. The estradiol and progesterone levels were low in non-pregnant women and increased gradually as pregnancy proceeded and doubled in late-pregnant women as of early-pregnant women (*P* < 0.001), as previously reported [24, 29]. The estradiol level in early-pregnant women was significantly higher than non-pregnant women (*P* < 0.001), while the progesterone level varied slightly (*P* = 0.0418) (Fig. 3a and b). Increasing estradiol and progesterone level shared the same variation trend with circulating ILC2s, which also reached peak levels in late-pregnant women (Table 2). Since previous studies show that sex hormones have a positive regulatory effect on ILC2s in murine uterus [43], to further understand the association of sex hormone level with ILC2s in human peripheral blood, we employed the Spearman’s correlation analysis and found that the proportion of circulating ILC2s either in total ILCs or in CD45^+^cells was positively correlated both with estradiol (*P* < 0.001) and progesterone (*P* < 0.001) (Table 3). Our results suggest that the dynamical distribution of circulating ILC subsets may be a result of physiological changes of plasma estradiol and progesterone level during pregnancy.

**Table 2:** Levels of circulating ILC2, estradiol, and progesterone

	Non-pregnant	Early-pregnant	Late-pregnant	*P* value
*N*	25	25	25	
ILC2				
Percentage in ILCs, %	36.40 (29.90, 46.90)	55.80 (48.30, 57.50)	77 (72.20, 79.10)	< 0.001***
Percentage in CD45^+^ cells, %	0.02 (0.02, 0.04)	0.04 (0.03, 0.06)	0.07 (0.05, 0.12)	< 0.001***
Absolute count, per ml	98.81 (64.02, 186.15)	191.22 (113.02, 298.87)	289.61 (229.21, 397.14)	< 0.001***
Estradiol, pg/ml	57.10 (34.16, 88.56)	408.48 (297.93, 488.08)	6362.75 (5534.62, 7410.15)	< 0.001***
Progesterone, ng/ml	4.61 (3.24, 10.22)	13.77 (10.70, 21.26)	164.37 (108.61, 214.33)	< 0.001***

Variables are shown as median (Q1, Q3). Comparisons of levels of circulating ILC2s, plasma estradiol, and progesterone among non-pregnant, early-pregnant, and late-pregnant women were assessed using Kruskal-Wallis tests. *P*-values less than 0.05 (*P* < 0.05) were considered significant. ***P < 0.001. *Q1* the first quartile, *Q3* the third quartile

**Table 3:** Spearman correlations among circulating ILC2, estradiol, and progesterone

	Estradiol	Progesterone
Percentage in ILCs	0.7933^***^	0.6752^***^
Percentage in CD45^+^ cells	0.5876^***^	0.4620^***^
Absolute count per ml	0.5915^***^	0.4638^***^

***Correlation is significant at the 0.001 level (2-tailed)

### Estrogen and Progesterone Receptors in Circulating ILC2s Change Differently Before and During Pregnancy

**Fig. 4:**
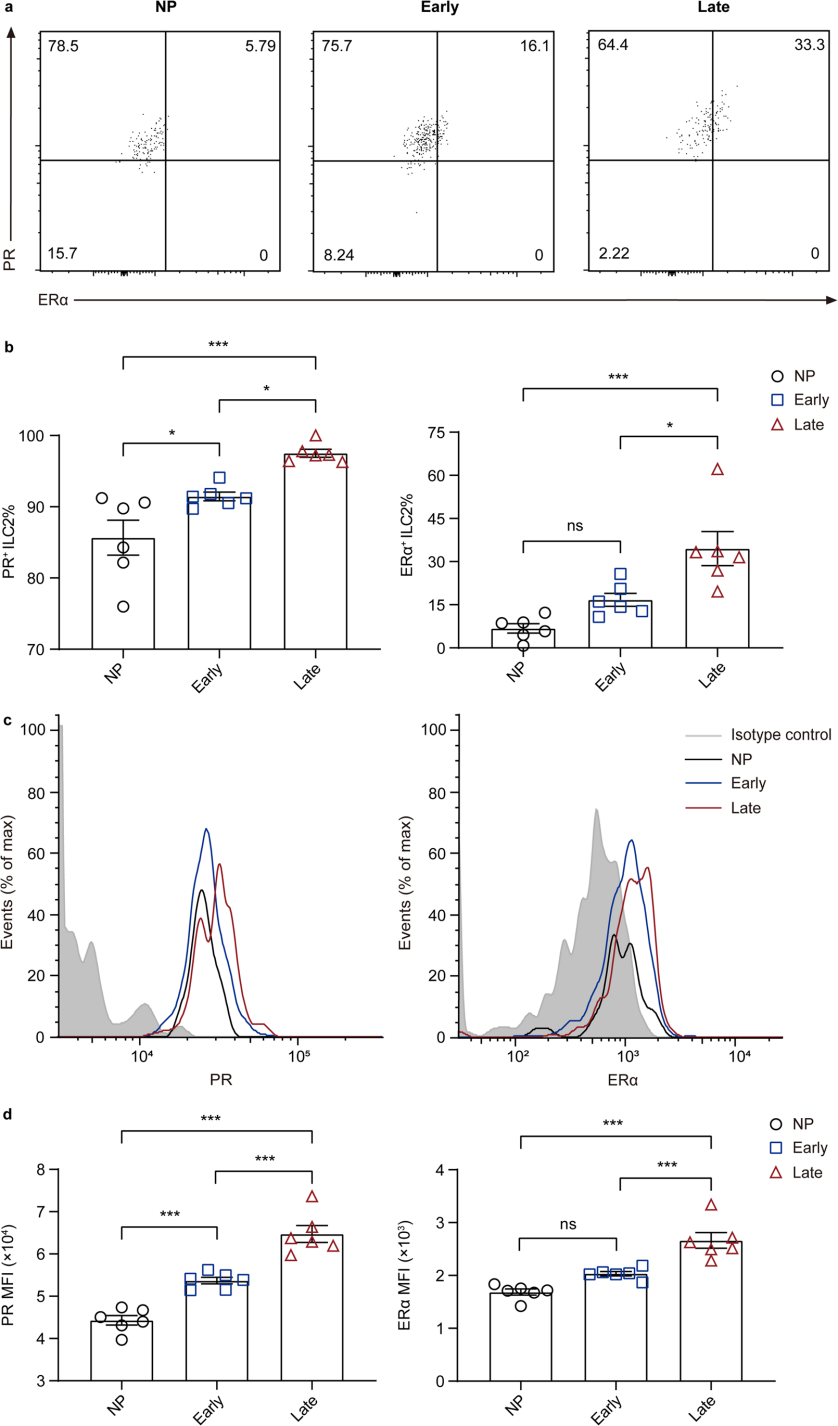
The estrogen and progesterone receptors in circulating ILC2s change differently in non-pregnant and pregnant women of different trimesters. **a–d** Flow cytometric analysis for PR and ERα in peripheral ILC2s of non-pregnant, early-pregnant, and late-pregnant women. **a** Representative flow cytometry plots are shown in which numbers indicate the frequency of flow cytometric events. **b** Comparison of and PR^+^ ILC2 and ERα^+^ ILC2 proportion in peripheral blood of non-pregnant women (black circles), early-pregnant women (blue squares), and late-pregnant women (red triangles). **c** Representative flow cytometry histograms of intracellular PR and ERα in circulating ILC2s from isotype control (grey area), non-pregnant women (black lines), early-pregnant women (blue lines), and late-pregnant women (red lines). **d** Quantification of PR and ERα mean fluorescence intensity (MFI) of circulating ILC2s. Data are shown as means ± SEMs and were analyzed by one-way ANOVA. *P < 0.05 and ***P < 0.001. *ns*, not significant

Sex hormones regulate physiological processes via their receptors. To further clarify the association of plasma estradiol and progesterone with circulating ILC2s, we randomly chose six women in each study group and analyzed the ERα, which is highly expressed in ILC2s of murine uterus [43] and progesterone receptor (PR) levels in ILC2s (Table S1). We found that in peripheral blood, ILC2s expressing ERα were also PR^+^ (Fig. 4a). Notably, the percentage of ERα^+^ ILC2 in late-pregnant women was significantly higher than early-pregnant (*P* = 0.0120) and non-pregnant women (*P* < 0.001), while no statistical difference was found between early-pregnant and non-pregnant women (*P* > 0.05) (Fig. 4a and b). In addition, a higher percentage of PR^+^ ILC2 was observed in late-pregnant women than early-pregnant (*P* = 0.0297) and non-pregnant women (*P* < 0.001), and the ratio differed slightly between early-pregnant and non-pregnant women (*P* = 0.0380) (Fig. 4a and b). Furthermore, examination of intracellular PR and ERα expression showed patterns of differential expression in ILC2s, in which PR and ERα expression by late-pregnant samples was much higher to that by non-pregnant and early-pregnant samples (both *P* < 0.001) (Fig. 4c and d). The observation of differential hormone receptor expression in circulating ILC2s among non-pregnant, early-pregnant, and late-pregnant women indicates the association of estradiol and progesterone with ILC2s in human peripheral blood.

## Discussion

ILCs have been reported as a new population of innate immune cells and regarded as the innate equivalents of T cells [13, 48]. Emerging from the lymphoid lineage but do not express antigen-specific receptors or undergo clonal selection [49], ILCs react rapidly to signals from injured sites and produce corresponding cytokines, which eventually lead to multiple immune pathways. According to the diverse expression of transcriptional factors, helper ILCs have been divided into three subsets — ILC1s, ILC2s, and ILC3s, which are counterparts of T helper cells. ILCs are widely distributed in mucosal and non-mucosal tissues, including the skin, intestines, lungs, spleen, and secondary lymphoid tissues [13, 48]. Here we analyzed the proportion of total ILCs and each ILC subset in total ILCs in peripheral blood of non-pregnant and pregnant women of different trimesters and demonstrated that the proportion of ILCs in peripheral blood of late-pregnant women was statistically higher than early-pregnant and non-pregnant women. Furthermore, the proportion and absolute counts of circulating ILC2s were significantly higher in late-pregnant women than early-pregnant and non-pregnant women, while ILC3s were much lower. Compared to non-pregnant women, the proportion of circulating ILC2s in early-pregnant women increased slightly, and no difference was found in the proportion of ILC3s. These results appear to align with previous reports about human decidua [12, 16, 19]. Additionally, we found that the middle trimester seemed to be the transitional period for dynamical changes of circulating ILC subsets (Table S2, Fig. S1). Based on our results, we hypothesized that helper type ILCs might be involved in maintaining normal pregnancy by changing the distribution pattern in different trimesters.

Sex hormones are critical to a healthy pregnancy and can modulate the distribution of immune cells. To determine the factors that contributed to the dynamic distribution of ILC subsets in peripheral blood of non-pregnant and pregnant women of different trimesters, we detected plasma estradiol and progesterone levels. Agreed with previous studies, we showed that the levels of plasma estradiol and progesterone were increased along with pregnancy proceeds and reached peak value during late pregnancy [24, 25], which was consisted with the variation trend of circulating ILC2s. Spearman’s correlation analysis demonstrated positive correlation between sex hormone levels and ILC2 proportion, which suggests that dynamical distribution of circulating ILC subsets during pregnancy may be a result of physiological elevating of plasma estradiol and progesterone level. The higher expression of estrogen and progesterone receptors in circulating ILC2s of late-pregnant women provide prima facie evidence for our hypothesis. Being well studied, estradiol in human and mice is recognized to regulate both adaptive and innate immune cells [24–28]. A recent study has shown that ILC2s in murine uterus can be regulated by estradiol through ERα [31]. Another previous research of murine ILC2s in different tissues indicates that ILC2s express a narrow panel of sex hormone receptors while progesterone receptor is barely expressed [37]. In human, progesterone is reported to favor the development of Th cells producing Th2-type cytokines [29], the counterparts of ILC2s. However, the progesterone receptor in human ILC2s is poorly studied. We observed the expression of progesterone receptors in human circulating ILC2s for the first time. Since sex hormone receptors are nuclear transcription factors as well as modulators of cell signaling pathways [25], our results suggest a potential role of plasma sex hormones regulating ILC2s in human peripheral blood.

In view of the fetus being a semiallograft, maintaining the balance of immune system is of great importance to a successful and healthy pregnancy. The critical roles of T cells as well as NK cells in a successful pregnancy have been demonstrated in lots of studies [1, 4, 17, 50], while the exact roles of helper type ILCs during pregnancy remain unknown. There was some research about uterine and decidual ILCs [8, 10, 12, 16, 17, 19, 22, 50], but the origin of these ILCs is still undetermined. A recent study considers ILCs as tissue-resident immune cells [51], the other one has found that circulating ILCs are immature ILCs [46], and the mechanism under which ILCs circulate between peripheral blood and tissues are still uncertain. Based on the above, we hypothesized that ILCs in peripheral blood may be the source of those in tissues, which needs further investigation. Although ILCs in maternal-fetal compartments have been studied over the years, the distributions of ILC subsets in peripheral blood during pregnancy are barely explored. Our data reveal the dynamic changes and distribution of circulating ILC subsets during normal pregnancy and the underlying role of estradiol and progesterone that modulates the proportion of ILC subsets.

Compared with the first trimester with a high risk of miscarriage [52] and the third trimester, which is about to undergo parturition, the second trimester seems to be a relatively stable state. Therefore, in terms of changes during pregnancy, we mainly focus on early and late pregnancy, which, in our opinion, were the most representative periods and better to show the variation of ILCs in peripheral blood. Furthermore, by detecting estradiol and progesterone receptors on circulating ILC2s in non-pregnant and pregnant women, our study finds a way for sex hormones to influence ILC distribution. However, our study does have some limitations that must be deliberated. The direct evidence of sex hormones regulating ILC subsets was insufficient, in vitro experiments are required. In addition, how circulating helper type ILCs play their roles in pregnancy, for example, effector cytokine secretion, also needs to be further studied, and the distribution pattern during pathological pregnancy is worthy of exploring.

In conclusion, this study demonstrated that in different trimester of normal pregnancy, despite NK cells, circulating helper type ILC subsets distributed diversely, as the level of ILC2s gradually increased along with pregnancy proceeded while ILC3s decreased. Elevating dramatically as pregnancy proceeded, estradiol and progesterone levels agreed with the variation trend of circulating ILC2s and might regulate ILC distribution via their receptors. We hypothesized that the dynamic changes of circulating ILC subsets might be adaptive feedback of pregnancy and are involved in fetal-maternal tolerance. Furthermore, increasing sex hormones may modulate transcriptional activity, thereby influencing the distribution of ILC subsets.

## Supplementary Information

ESM 1 (PDF 371 kb)

## Data Availability

The data that support the findings of this study are available from the corresponding author upon reasonable request.

## Abbreviations

ILCs: Innate lymphoid cells
PBMCs: Peripheral blood mononuclear cells
ILC1: Type 1 innate lymphoid cells
ILC2: Type 2 innate lymphoid cells
ILC3: Type 3 innate lymphoid cells
NK cells: Natural killer cells
Th cells: T helper cells
E1: Estrone
E2: Estradiol
E3: Estriol
PE-Cy7: R phycoerythrin-cyanine 7
PE: Phycoerythrin
ERα: Estrogen receptor α
RIA: Radioimmunoassay
BMI: Body mass index
PR: Progesterone receptor
NP: Non-pregnant women.
