# Supplementary material for: Circulating Innate Lymphoid Cells Exhibit Distinctive Distribution During Normal Pregnancy
Source: Reprod Sci. 2022 Jan 5;29(4):1124–35. doi: 10.1007/s43032-021-00834-6 (PMC8907087; doi:10.1007/s43032-021-00834-6)
Supplement: Supplementary file 1 — (PDF 371 kb) [file 43032_2021_834_MOESM1_ESM.pdf]

Circulating Innate Lymphoid Cells Exhibit Distinctive Distribution During Normal Pregnancy

Reproductive Sciences

Authorship

Yiran Zhao [a, b, †], Yajie Zhu [a, b, †], Xi Chen [a, b], Hui Lin [a, b], Ningxin Qin [a, b], Zhiyang Zhou [a, b], Han Liu [a, b], Yanhui Hao [a, b], Chengliang Zhou [a, b], Xinmei Liu [b, c], Li Jin [b, c], Jianzhong Sheng [d], Hefeng Huang [a, b, c, *]

a, the International Peace Maternity & Child Health Hospital, School of Medicine, Shanghai Jiao Tong University, Shanghai 200030, China

b, Shanghai Key Laboratory of Embryo Original Diseases, Shanghai 200030, China

c, Hospital of Obstetrics and Gynecology, Fudan University, Shanghai 200010, China

d, Department of Pathology and Pathophysiology, School of Medicine, Zhejiang University, Zhejiang 310058, China

†, These authors contributed equally to this work.

***,** Corresponding author: He-Feng Huang, huanghefg@sjtu.edu.cn, (86)021-64073897

Supplemental information

Table S1. Participant characteristics

Characteristic	Non-pregnant	Early-pregnant	Late-pregnant	*P* value
**N**	6	6	6	
**Age,** mean (SD), years	30.17 (4.02)	28.67 (1.37)	31.83 (3.06)	0.2254
**Pre-pregnancy BMI,** mean (SD), kg/m^2	20.31 (1.53)	19.67 (0.73)	20.53 (2.34)	0.6600
**Gravidity**, n (%)				0.0611
0	3 (50)	0 (0)	0 (0)	
1	1 (16.67)	1 (16.67)	3 (50)	
≥2	2 (33.33)	5 (83.33)	3 (50)	
**Parity**, n (%)				0.5467
0	3 (50)	2 (33.33)	4 (66.67)	
1	1 (16.67)	2 (33.33)	2 (33.33)	
≥2	2 (33.33)	2 (33.33)	0 (0)	
**Previous induced abortions**, n (%)				0.0069 **
0	5 (40)	0 (44)	0 (64)	
1	0 (44)	3 (40)	3 (16)	
≥2	1 (16)	3 (16)	3 (20)	
**Gestational age**, median (Q1, Q3), weeks	/	6 (6, 6.75)	39.15 (38.57, 39.40)	0.0022 **

Comparisons of characteristics among non-pregnant, early- pregnant and late-pregnant women were assessed using one-way ANOVA or Mann-Whitney test for continuous variables and Chi-squared tests were used for categorical variables.

P-values less than 0.05 (P<0.05) were considered significant. ** P <0.01.

BMI, body mass index. SD, standard deviation. Q1, the first quartile. Q3, the third quartile.

Table S2. Participant characteristics

Characteristic	Non-pregnant	Early-pregnant	Mid-pregnant	Late-pregnant	*P* value
**N**	25	25	22	25	
**Age,** mean (SD), years	30 (3)	29.72 (2.31)	29.23 (3.13)	30.36 (2.66)	0.5610
**Pre-pregnancy BMI,**					
mean (SD), kg/m^2	20.12 (1.32)	20.21 (1.48)	20.88 (1.66)	20.39 (2.20)	0.4420
**Gravidity**, n (%)					<0.001 ***
0	7 (28)	0 (0)	0 (0)	0 (0)	
1	8 (32)	4 (16)	8 (36)	15 (60)	
≥2	10 (40)	21 (84)	14 (64)	10 (40)	
**Parity**, n (%)					0.0018 **
0	15 (60)	8 (32)	19 (86)	19 (76)	
1	4 (16)	9 (36)	3 (14)	5 (20)	
≥2	6 (24)	8 (32)	0 (0)	1 (4)	
**Previous induced**					0.2863
**abortions**, n (%)					
0	10 (40)	11 (44)	12 (54)	16 (64)	
1	11 (44)	10 (40)	9 (41)	4 (16)	
≥2	4 (16)	4 (16)	1 (5)	5 (20)	
**Gestational age,**					
median (Q1, Q3), weeks	/	6 (6, 6)	26.22 (24.86, 26.71)	39.14 (38.79, 39.79)	<0.001 ***

Comparisons of characteristics among non-pregnant, early-pregnant, mid-pregnant and late-pregnant women were assessed using Kruskal-Wallis tests or one-way ANOVA or Mann-Whitney test for continuous variables and Chi-squared tests were used for categorical variables.

P-values less than 0.05 (*P*<0.05) were considered significant. * *P* < 0.05 and *** *P* <0.001.

BMI, body mass index. SD, standard deviation. Q1, the first quartile. Q3, the third quartile.

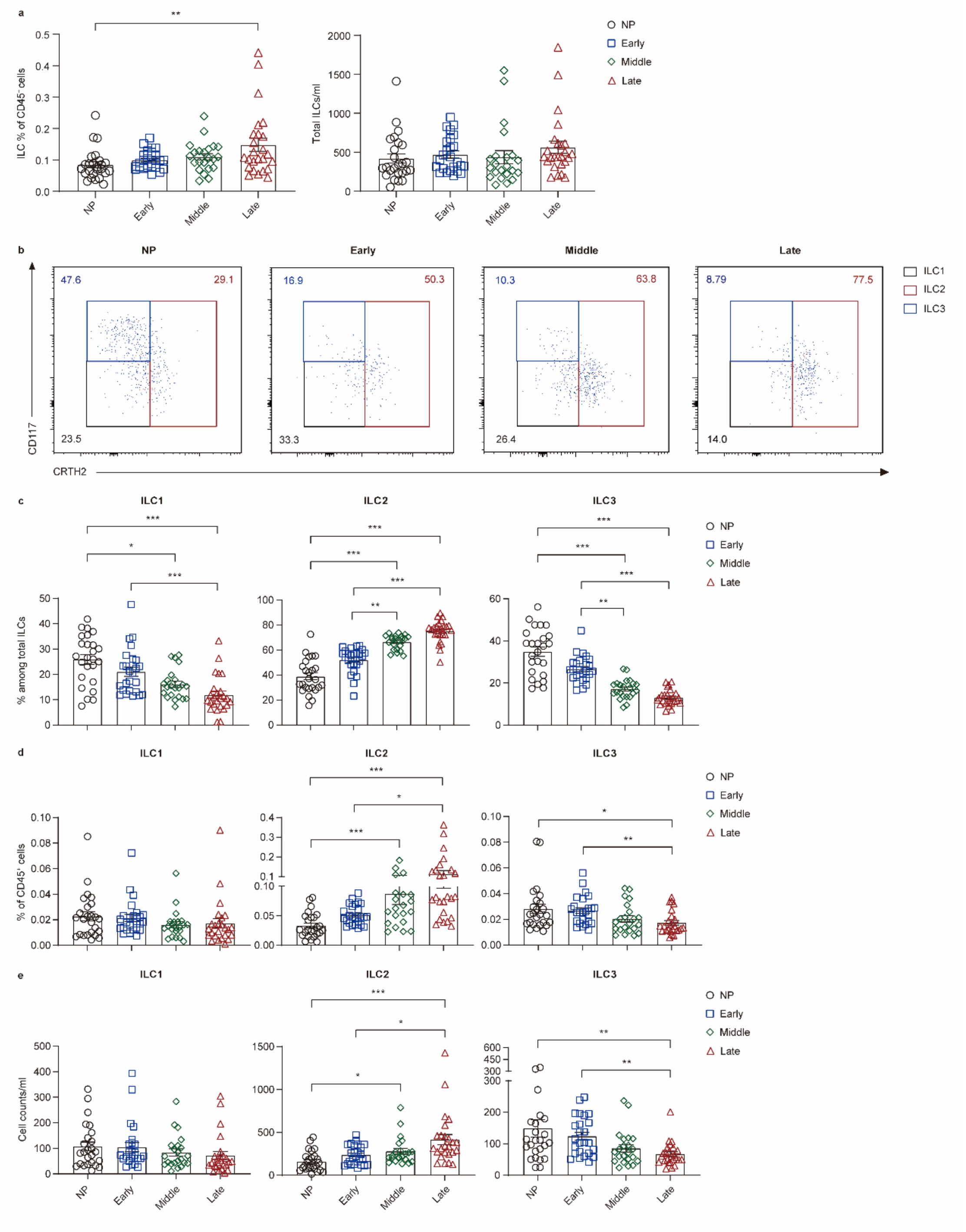

Fig S1. The middle trimester shows transitional change of circulating ILC subsets. **(a-e)** Analysis results for ILC subsets in peripheral blood samples of non-pregnant, early-pregnant, mid-pregnant and late-pregnant women. **(a)** Comparison of circulating ILC percentage in $CD45^+$ cells (left) and absolute counts (right) in non-pregnant, early-pregnant, mid-pregnant and late-pregnant women. **(b)** Representative flow cytometry plots are shown in which numbers indicate the frequency of flow cytometric events. Comparison of proportion of ILC subsets within total ILCs **(c)**, ILC subset percentage in $CD45^+$ cells **(d)**

and absolute counts per ml blood **(e)** in peripheral blood of non-pregnant women (black circles), early-pregnant women (blue squares), mid-pregnant women (green diamonds) and late-pregnant women (red triangles). Data are shown as means ± SEMs and were analyzed by Kruskal-Wallis tests. Each point indicates an individual. $^{*}P < 0.05$, $^{**}P < 0.01$ and $^{***}P < 0.001$.
